# The Fundamental and Underrated Role of the Base Electrolyte in the Polymerization Mechanism. The Resorcinol Case Study

**DOI:** 10.1021/acs.jpca.0c07702

**Published:** 2020-12-22

**Authors:** Marco Bonechi, Massimo Innocenti, Davide Vanossi, Claudio Fontanesi

**Affiliations:** † Department of Chemistry, University of Firenze, via della Lastruccia 3, 50019 Sesto Fiorentino, Italy; ‡ Department of Engineering “Enzo Ferrari”, University of Modena and Reggio Emilia, Via Vivarelli 10, 41125 Modena, Italy

## Abstract

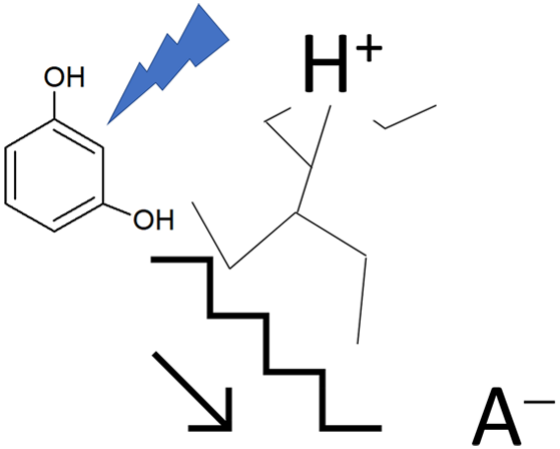

The Kane–Maguire polymerization mechanism is disassembled at a molecular level by using DFT-based quantum mechanical calculations. Resorcinol electropolymerization is selected as a case study. Stationary points (transition states and intermediate species) leading to the formation of the dimer are found on the potential energy surface (PES), and elementary reactions involved in the dimer formation are characterized. The latter allow to further propagate the polymerization chain reaction, when applied recursively. In this paper, the fundamental role of the sulfate anion (a typical base electrolyte) is addressed. Investigation of the PES in terms of both stationary-state properties and of ab initio molecular dynamics results (dynamic reaction coordinate) allows the appreciation in detail of the critical role of the base electrolyte anion in making the proton dissociation from the initial radical ion, a feasible (downhill in energy) process.

## Introduction

Chemistry is well-known as a complex, multifaceted, and nonlinear science,^1^ spanning from pure compound characterization to the field of “chemical reactivity”. In particular, the knowledge of chemical reactivity is somehow the ultimate goal of a chemist, where even “experimentalists” do not limit their activity to pure laboratory/instrumental work, but they do love to speak of “reaction mechanism”.^2^ The latter is a dramatically complex subject, quite often mixing physics and chemistry in a nonlinear way: the ultimate achievement is writing the reaction “time law”, which is expressed by a system of partial differential equations.^2,3^ In this respect, electrochemistry is an archetypal field of scientific research where the reductionist approach (here reductionist is meant as an elementary scheme focusing only on the properties of the molecule involved in the reaction and ignoring complicating side processes) can lead to interesting results, concerning both faradaic processes (reduction/oxidation) and “simple” electrochemical-driven adsorption/desorption interfacial processes.^4−10^ Within such an approach, the role of the base electrolyte is in general neglected, as the ideal base electrolyte is both hoped and thought to simply allow for electrical conductivity in solution without participating in any chemical rection. For instance, a reductionist approach allows the rationalization in detail of the first electron transfer in faradaic processes: for instance, the well-known LUMO energy vs reduction potential relationship.^9^ Also, in the case of dissociation reactions following the first electron transfer, a reductionist reaction scheme is able to catch the energy/“molecular structure” relationship. But this is not the case when a series of complex chemical reactions follow the first charge transfer process, for instance, in the case of electropolymerization reactions, where ample studies are devoted to the determination of the properties of the polymer rather than to the reaction mechanism (which is by far a more complex subject).^11,12^ In this field a detailed and successful reaction mechanism rationalization based on only the characteristics of the pristine compounds does not work.^13^ Thus, in this paper the focus is on the resorcinol polymerization mechanism, which is a quite important chemical reaction, also in connection with applicative aspects connected to the production of industrial resins.^14−17^ In fact, resorcinol polymerization must proceed through the dissociation of one (or two) hydrogen atom, as it is also assumed in resorcinol condensation reactions.^18^ This is the case for other complex reactive systems, spanning from photochemistry to electrochemistry, where the charge transfer elementary act is coupled with the proton dissociation.^19−22^ In our study we focused on the initial reaction steps leading to the formation of dimers. Dimers can be produced following (i) the formation of a carbon–oxygen bond, (ii) the formation of a carbon–carbon bond, and (iii) the rather exotic, but possible, formation of a peroxo compound. In any case the polymerization reaction must proceed with the dissociation of an O–H or C–H bond. In this paper a theoretical approach is followed to shed light on the polymerization elementary mechanism, with a detail which is experimentally out of reach. To study the polymerization mechanism, we relied not only on a steady-state potential energy surface analysis (determining stationary points as equilibrium reagents, products, intermediate species, and transition states) but also on ab initio molecular dynamics (MD) calculations by using the dynamic reaction coordinate (DRC) approach.^23^ The latter is a classical trajectory analysis based on an ab initio PES (as electronic state description cannot be avoided in the case of reactions involving radical charged species). It is clearly shown that the presence of the base electrolyte, namely, here the sulfate anion, SO_4_^2–^, is of fundamental importance making the dissociation of the H^+^ feasible. Thus, this shows that the SO_4_^2–^ is acting as a sort of catalyzer decreasing dramatically the dissociation energy of the O–H or C–H process, eventually making it a feasible reaction.

## Methods

All the theoretical results here reported concerning species in all possible oxidation states and spin multiplicity are performed in the framework of ab initio quantum mechanical based methods. Unless otherwise indicated, all calculations were performed using *C*1 symmetry and unrestricted wave function, by using the GAMESS,^24^ Gaussian 16,^25^ and Firefly Rev 8.20^26^ (Firefly is partially based on the GAMESS (US)8 source code) programs. Chemcraft^27^ is used for visualization purposes, both molecular structures, and ab initio molecular orbitals display, and MacMolPlt^28^ served to display DRC trajectories. Original Fortran-based codes were created for the extraction of molecular geometrical parameters from DRC calculations, to allow for the analysis of angle and bond distance variations as a function of time (available on request from the author C.F.). For all the structures reported as stationary states shown in the PES versus reaction coordinates plot, reaction paths A, B, C, and D, *vide infra*, molecular geometries are obtained by full optimization carried out at both the UB3LYP/6-31G* and UB3LYP/cc-pVTZ levels of the theory. To account for solute–solvent interaction, geometry optimization is carried out by using the Barone and Cossi’s polarizable conductor model (CPCM);^29,30^ the latter is based on Tomasi’s Polarized Continuum Model (PCM).^31^ The stability of all the species is checked by Hessian calculation (vibrational frequency spectrum). In the case of reagents, products, and intermediate reaction species, all the frequency values are found as real and positive. Transition state search was pursued by analyzing relaxed scan curves. Transition states feature a single imaginary negative frequency, and two negative frequencies are found for the intermediate species which dissociates two protons to yield the final dimer.

In ab initio molecular dynamics, DRC trajectories (as implemented in the GAMESS and Firefly programs) are started at molecular geometries relevant to stationary points on the reaction PES, i.e., transition states and intermediate reaction species. The velocity vector needed to start the molecular dynamics is obtained by the projection of Hessian vibrational eigenvectors. In the present DRC results the kinetic energy is partitioned over all normal modes, assigning only the zero-point energy to each normal mode (unless otherwise stated). This attempts to minimize the influence of the initial geometrical and velocity vector on the molecular dynamic trajectory (an alternative strategy would be to assign arbitrary energy to some selected vibrational modes, for instance the mode leading to dissociation of the proton which corresponds to an imaginary frequency in the transition state).^32^ MP3/cc-pVTZ and MP4(SDTQ)/cc-pVTZ single-point energy calculations (Figure S1) are used to cross-check the energy ordering of the DFT data (Møller–Plesset postscf method exploits a completely “different” strategy to account for electron correlation effects). This is a point of paramount importance in that DRC calculations that are run on a “wrong” PES would be meaningless.

## Reaction Mechanism

**Scheme 1:**
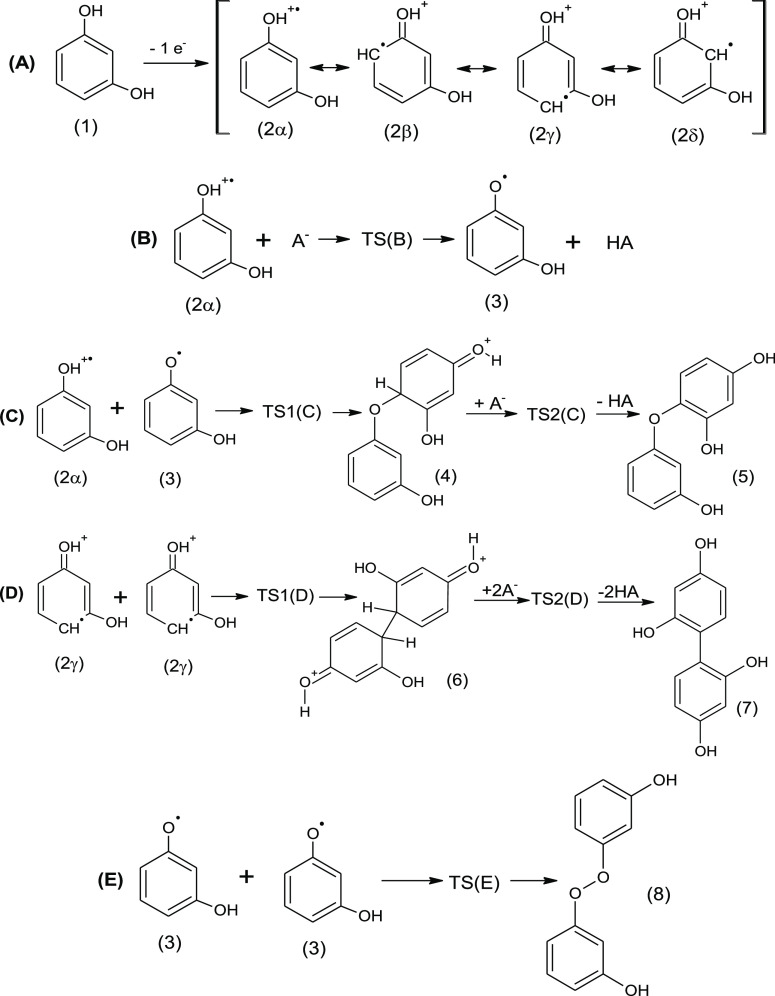


Scheme 1 shows the single elementary steps considered in the polymerization of resorcinol. The elementary steps are mainly inspired by the Kane–Maguire mechanism.^33,34^

Elementary reaction steps of five different reaction paths are considered. Path A refers to the simple one-electron oxidation of resorcinol (**1**) leading to the relevant radical cation (**2**); in principle this is a reversible step. Suitable experimental conditions, low temperature, very fast potential scan in cyclic voltammetry (CV), and ultradry and oxygen free solvent, could allow for the observation of a reversible or quasi-reversible voltammogram.^35,36^ The standard oxidation potential, , of the **1**/**2** redox couple has been calculated at the UB3LYP/cc-pVTZ, resulting in +1.1 V vs Ag/AgCl/KCl_sat_ (compare the Supporting Information for the calculation details), which is a result in fair agreement with the experimental value.^37^

Path B is one possible main route for the polymerization reaction, leading to the neutral radical species **3** by the dissociation of the hydroxylic O–H proton: the formation of **3** is a crucial reactive species in the possible polymerization propagation mechanism.

The simplest follow-up reaction involves radicals **2** and **3**, path C:

Path C yields a stable closed-shell dimer, **5**, through intermediate charged species **4**. Remarkably, a proton dissociation reaction, **4** to **5** via transition state TS2(C), is the crucial step. We will address explicitly the role of A^(−)^.^38^

The Path D final product is the closed-shell dimer species **7**. In this case the most complex step is the dissociation of two protons of intermediate species **6**. The protons are yielded by C–H bond dissociation.

Path E is considered for the sake of a complete approach. Direct reaction involving two neutral radicals, **3**, with the formation of the closed shell dimer **8** (Supporting Information section Peroxo Product). Indeed, **8** appears as a rather reactive unstable peroxide.

## Results and Discussion

### 1 Reaction Paths A and B

**Figure 1:**
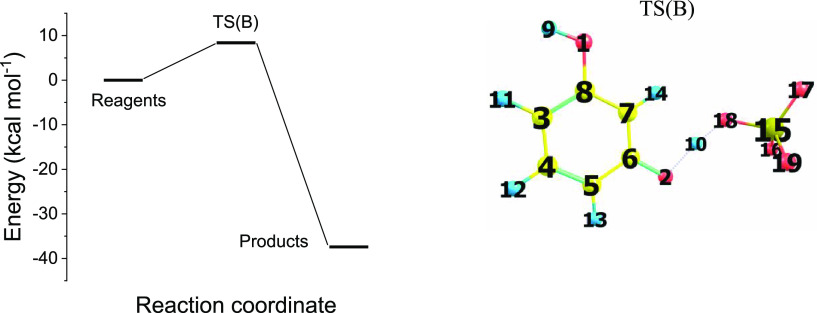
Potential energy surface for reaction path B, at the B3LYP/cc-pVTZ level of theory. Gibbs free energy of reagents , final products , and transition state (TS(B)). Bonds used to characterize the proton dissociation mechanism: O_2_–H_10_.

A detailed analysis of the polymerization mechanism clearly shows how, following the oxidation process (mechanism step A, *vide supra*), a fundamental role is played by the dissociation of the proton from the resorcinol radical cation (mechanism step B, the elementary step leading to TS(B)). Or, an alternative route is the dissociation of two protons in the intermediate species, **6**, in path D, which eventually yields a dimer, 7, through the formation of a new single C–C bond. Indeed, the direct proton dissociation is revealed to be a nonviable process because the relaxed dissociation scan of species **2** shows an uphill energy pattern. The dissociation energy of **1** is found to be larger than 100 kcal mol^–1^, including an explicit water molecule with a relaxed scan in which the dissociation energy decreases to 32 kcal mol^–1^, which still (at 298 K) is an energy barrier too large for allowing a spontaneous proton dissociation (Supporting Information Figure S2). A completely different picture is obtained when the base electrolyte anion, in the case of the sulfate (SO_4_^2–^) anion, is considered. Figure S3 shows the relaxed-scan PES concerning the elementary reaction , i.e., the proton dissociation assisted by the sulfate anion. The overall process is downhill in energy. Figure 1 shows the path B stationary-state PES; the right panel in Figure 1 sketches the TS(B) structure. TS(B) is found to be 8 kcal mol^–1^ higher in energy than the reagents; thus, the dissociation process features a feasible activation energy, and from the thermodynamic point of view the path B process is 37 kcal mol^–1^ more stable in energy going from reagents (**2** + SO_4_^2–^) to products (**3** + HSO_4_^–^); compare Figure 1.

### 2 Path C

**Figure 2:**
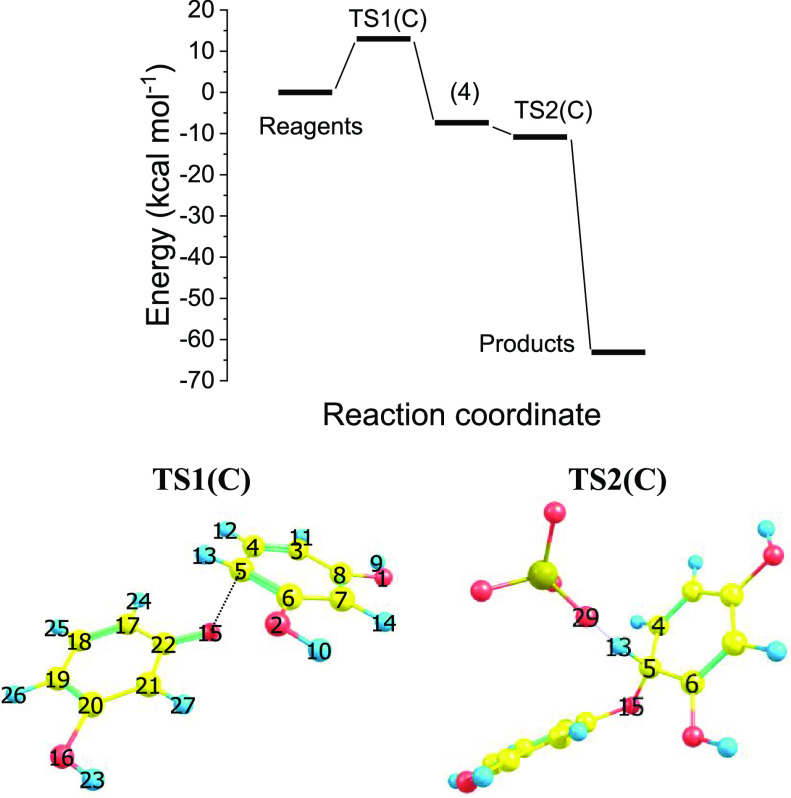
Potential energy surface for reaction path C, at the B3LYP/cc-pVTZ level of theory. Gibbs free energy of reagents , final products , reaction intermediate , and transition states  and TS2. Bonds used to characterize the proton dissociation mechanism: H_13_–O_29_, C_5_–O_15_, C_5_–H_13_. TS1(C) and TS2(C) dihedral angle atoms 6–4–5–13 are used to characterize the proton dissociation mechanism.

Path B products can allow to propagate further into step C, where the reaction between **2** (resorcinol radical cation) and **3** (the relevant deprotonated species) takes place. The formation of the closed shell dimer **5** proceeds through the formation of transition state TS1(C) and the sigma complex intermediate species **4**. Then, the sigma complex deprotonation occurs due to the reaction with the sulfate anion: . This eventually yields the dimer **5**, where the two aromatic rings are bridged via a C_sp2_–O–C_sp2_ bond. Figure 2 shows the energy pattern of path C relevant to the five stationary-state systems: (i) reagents ; (ii) ; (iii) ; (iv) TS2(C); and (v) . Figure 2 shows also the transition states TS1(C)and TS2(C) molecular structures.

Path C could also yield the formation of a peroxo compound, where an O–O bond connects the two aromatic rings. This reaction path is due to the reaction between two **3** radicals (Supporting Information Figure S8).

### 3 Path D

**Figure 3:**
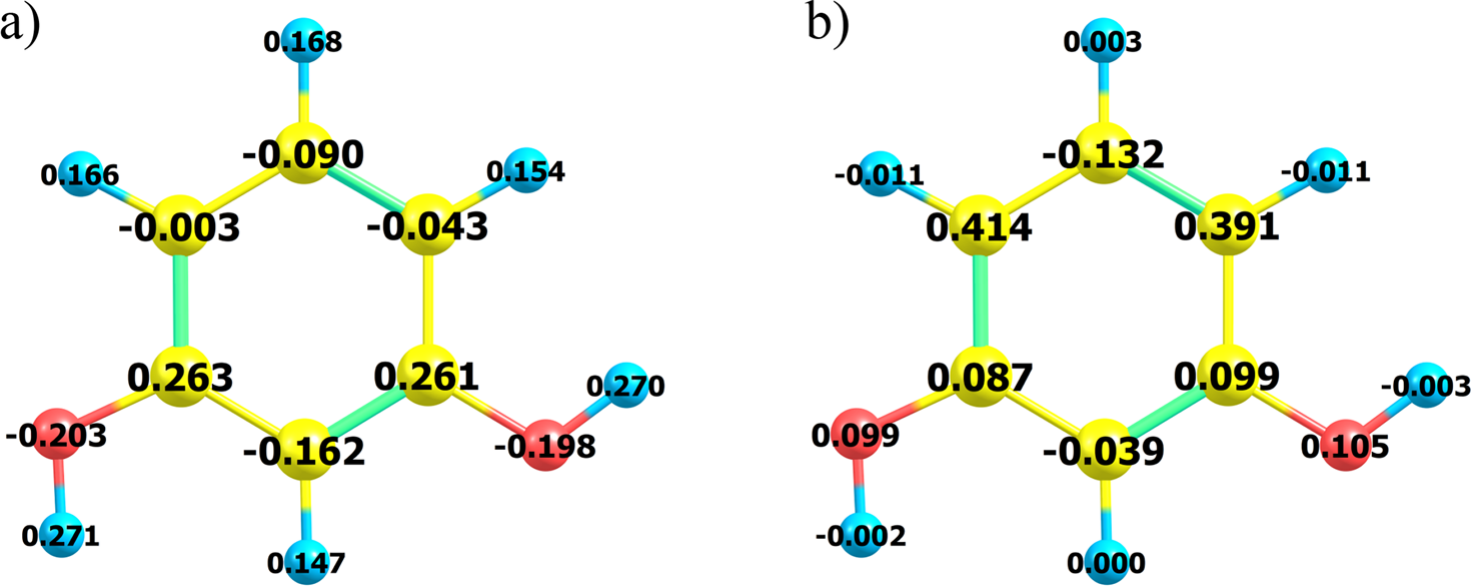
Resorcinol radical cation, B3LYP/cc-pVTZ: (a) Mulliken net charges; (b) atomic spin density distribution.

It is convenient, before going into the details of route D, to examine net charge and atomic spin density distribution concerning the radical cation **2** (Figure 3), aiming to discern the most probable reactive site of **2**. Indeed, the analysis of the mesomeric effect (resonance structures shown in the scheme relevant to path A, *vide supra*) shows that the position ortho to the hydroxylic moiety should be the most reactive. This is also supported by B3LYP/cc-pVTZ results, as the carbon net charge is the lowest (ensuring minimization in coulomb repulsion) and the atomic spin density is the highest (suggesting high reactivity in forming a new bond). This is in qualitative agreement with the results reported by Nady et al.^39^

**Figure 4:**
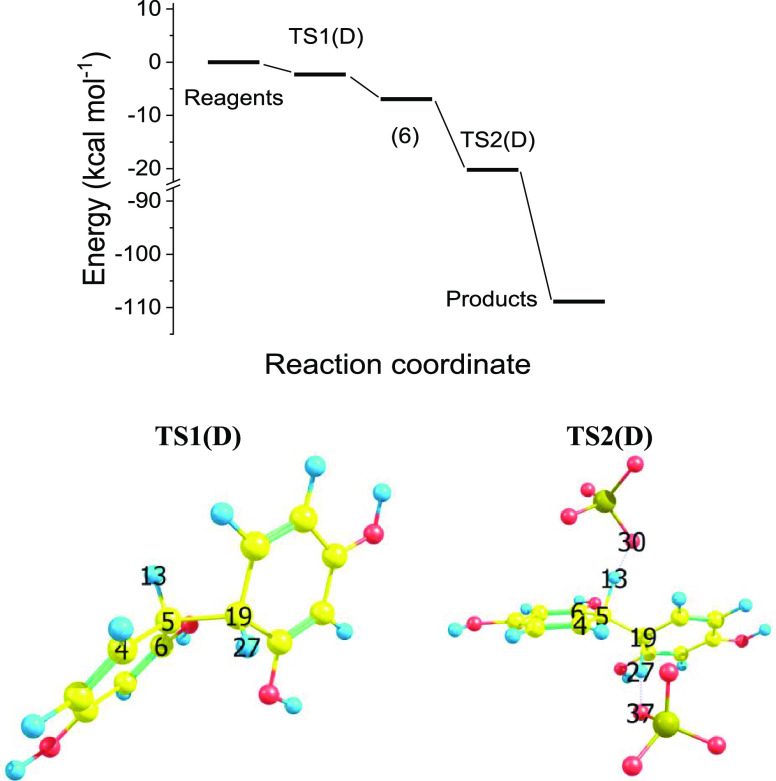
Reaction path D, at the B3LYP/cc-pVTZ level of theory. Gibbs free energy of reagents , final products , reaction intermediate **6**, and transition states  and TS2(D). TS1(D) and TS2(D) dihedral angle atoms 6–4–5–13. Bonds used to characterize the proton dissociation mechanism: H_13_–O_30_, H_27_–*O*_37_, C_5_–C_19_, C_5_–H_13_, C_19_–H_27_.

For path D here will be reported the results in detail concerning only the most reactive position as the “active” site for dimer formation through a single C_sp2_–C_sp2_ bond, which leads to intermediate species **6** and final dimer **7**. Indeed, different reactive paths have been considered too; for instance, the molecular structure relevant to an attempt of carbon hydrogen dissociation is reported in the Supporting Information (Figure S9). Route D features the direct coupling of radical cations, **2**, to produce the sigma complex dication (**6**). Hessian analysis of **6** following geometry full-optimization shows that it is a real minimum, all the frequencies being real and positive (both B3LYP/6-31G(d) and B3LYP/cc-pVTZ results). Then the dissociation of two protons is needed to obtain the dimer **7**. Also, in this case the direct dissociation from **6** is a process dramatically uphill in energy, just more than 100 kcal mol^–1^, while (as in the previous route C) the presence of sulfate anions explicitly considered causes a dramatic modification of the overall picture, and the  reaction becomes downhill in energy, as reported in Figure 4. Remarkably, route D is, basically, a monotonic step down in the energy reaction path, without any activation energy. This at variance with route C.

Once the dimer species **7** is obtained, the propagation of the chain reaction can occur by applying the same mechanism recursively. Note that path D can lead also to a stable quinoid-like structure, with the formation of a bond bridging the two carbon-based rings (the relevant molecular structure is reported in the Supporting Information, Figure S10). The formation of the quinoid-like dimer would stop the further propagation of the polymerization.

### 4 Molecular Dynamics Calculation: DRC for Path B, C, and D

Ab initio molecular dynamics calculations are calculated starting from different characteristic “points” of the PES. Namely, although not strictly required, in general DRC trajectories are started from reagents, reaction intermediate species, or transition states. The most important critical choice concerns the suitable selection of the velocity vector. In all the DRC results reported here, the kinetic energy is partitioned over each normal mode, and the initial velocity vector is obtained by projection of the Hessian matrix obtained by a previous calculation step, which is run at the initial DRC geometry. The initial kinetic energy is obtained assigning only the zero-point energy to each normal mode. Trajectory analysis focuses on the information concerning the variation of potential energy (dissociating-bond distances, dihedral angle variation) as a function of time. Trajectories as a function of time show a marked ripple which is due to the activity of the intramolecular/intermolecular vibrational mode.

#### Path B DRC

**Figure 5:**
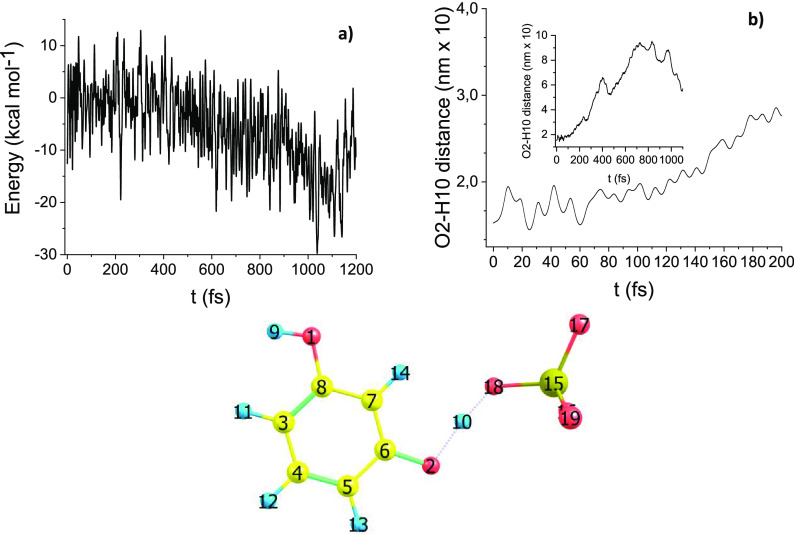
Path B DRC results, B3LYP/6-31G(d): (a) molecular electronic potential energy vs time; (b) O_2_–H_10_ bond distance vs time (inset in Figure 5b shows a wider time domain).

Figure 5a shows the potential energy versus time pattern obtained starting the trajectory from TS(B), route B. The potential energy decreases as a function of time, as can be expected based on previous PES vs reaction coordinate behavior, compared with Figure 1. Figure 5b displays the O_2_–H_10_ distance variation versus time, which is relevant to the dissociating proton. The proton dissociation, due to the presence of the sulfate group, becomes a feasible downhill process as shown in the previous section (compare Figure 1 and the relevant discussion). This is reflected in the overall DRC potential energy versus time trajectory. In Figure 5b for the oxygen proton distance as a function of time, after an initial induction period, the pattern is monotonically increasing from 60 fs onward.

#### Path C DRC

**Figure 6:**
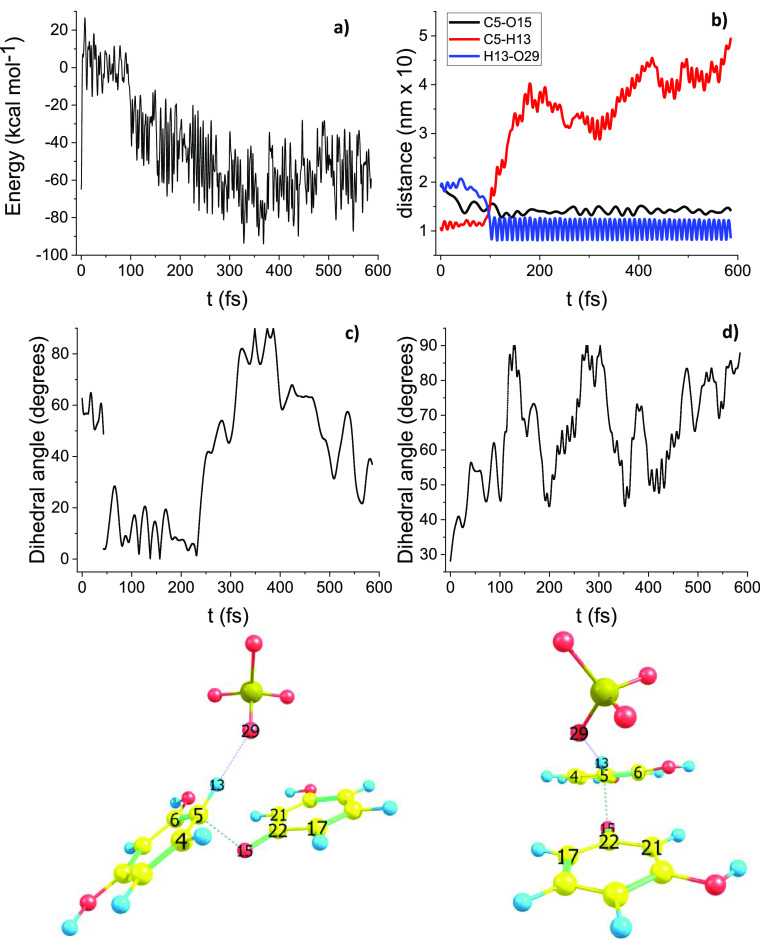
Path C DRC results, B3LYP/6-31G(d). (a) Molecular electronic potential energy vs time. (b) H_13_–O_29_, C_5_–O_15_, C_5_–H_13_ bonds distances. (c) Dihedral angle between rings, i.e., plane containing centers (4, 5, 6) and plane containing centers (17, 22, 21); molecular model on the right allows the appreciation of the relevant geometrical disposition. (d) Dihedral angle atoms 6–4–5–13; molecular model on the left allows the appreciation of the relevant geometrical disposition.

Figure 6 summarizes the DRC results pertinent to path C, which concerns the elementary reaction between the radical cations **2** and the relevant deprotonated neutral radical species **3**. The DRC starting point is the TS1(C) structure (sigma complex: C_5_ is coplanar with the five rings’ carbons, but it is sp^3^ hybridized). In this peculiar case two trajectories are calculated. Figure 6 shows the trajectory obtained with the explicit presence of the sulfate anion. Here the energy is monotonically decreasing as a function of time. Figure 6b shows that the H_13_–O_29_ distance, blue line, decreases from 2 to 0.9 Å (please note the regular oscillations after 50 fs, representative of the equilibrium H–O distance of the  moiety); a similar behavior is shown by the C_5_–O_15_ distance, black line, which decreases from 2 to 1.45 Å. In the same graph is also shown the dissociating bond C_5_–O_13_ distance, red, which after 100 fs increases monotonically.

Energy versus time and distance versus time patterns are marked by a discontinuity at about 100 fs for all these distance versus time graphs, Figures 6a and 6b, respectively, indicating a tight relation between the dissociating and forming chemical bonds. The DRC trajectory starting from TS1(C) without the presence of the sulfate anion is characterized by a constant potential energy versus time pattern, without any indication of proton dissociation from the relevant sigma complex (compare Figures S4 and S5, Supporting Information). Thus, the presence of the sulfate anion plays a decisive role in making the proton dissociation a feasible reaction.

#### Path D DRC

**Figure 7:**
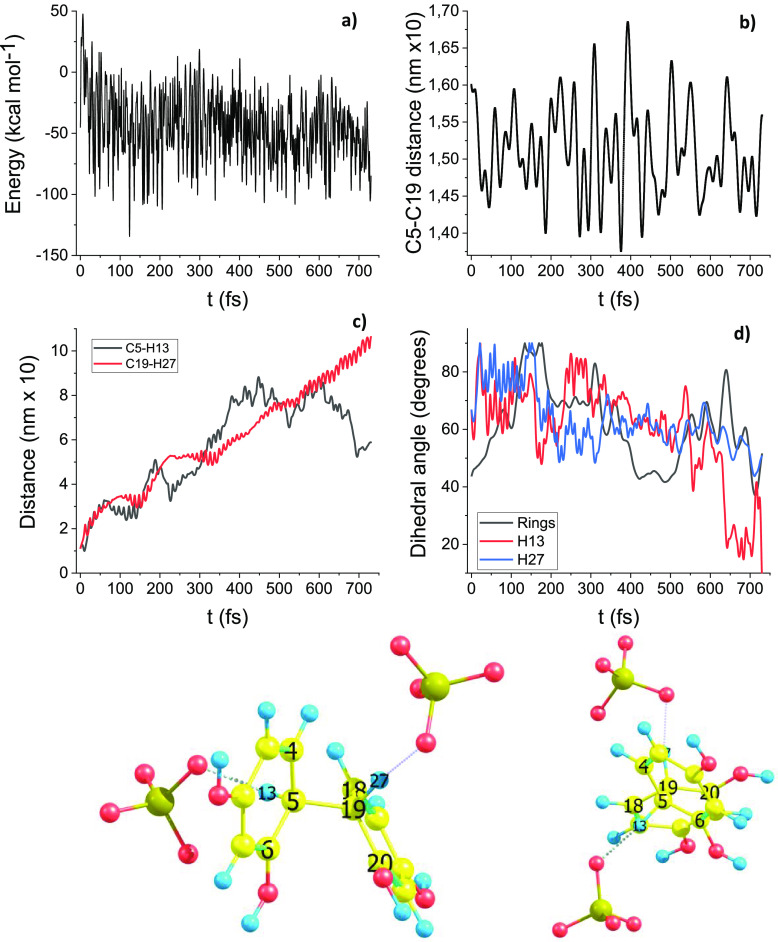
Path D DRC results, B3LYP/6-31G(d). (a) Molecular electronic potential energy vs time. (b) C_5_–C_19_ bond distance. (c) C_5_–H_13_ and C_19_–H_27_ bonds distances. (d) Dihedral angle between aromatic rings, i.e., plane containing centers (18, 19, 20) and plane containing centers (4, 5, 6); molecular model on the right allows the appreciation of the relevant geometrical disposition.

Figure 7 summarizes the DRC results pertinent to path D, maybe the most interesting. In that, the two aromatic rings form a dimer at the end of path D through a single C_sp2_–C_sp2_ bond. Here the elementary reaction between two radical cations **2** is considered. The starting point of the Figure 7 DRC trajectory is the TS1(D) structure, sigma complex **6**. In this peculiar case three DRC trajectories are calculated: Figure 7 shows the trajectory obtained with the explicit presence of two sulfate anions (total system charge is minus two; electronic state multiplicity is one). Also, in this case the energy is monotonically decreasing as a function of time. Figure 7b shows that the H_13_–C_5_ (black line) and H_19_–C_19_ (red line) distances increase from 1 Å to nonbonded distances. After 120 fs both these distances are larger than 2 Å. The DRC trajectory starting from TS1(D) without the presence of the sulfate anion is characterized by a constant potential energy versus time pattern, without any indication of proton dissociation from the relevant sigma complex (compare Figures S6 and S7, Supporting Information). Thus, also concerning path D the presence of the sulfate anion plays a decisive role in making the proton dissociation a feasible reaction. Eventually, a DRC calculation was started from TS2(D), and the relevant overall results fall in line, in tight agreement, with the DRC outcome of Figure 7a.

## Conclusions

For the first time the Kane–Maguire polymerization mechanism (for the resorcinol case study) has been disassembled and studied in terms of ab initio molecular dynamics and reaction elementary steps. To such a level of molecular detail, the theoretical approach remains the only viable approach. DFT steady-state and molecular dynamics results indicate that the sulfate anion plays a fundamental role, making the crucial first step of proton dissociation a feasible process: direct proton dissociation is about 100 kcal mol^–1^ uphill in energy. The proposed mechanism for the resorcinol electrochemical polymerization is fully consistent with experimental results present in the literature and with preliminary experimental results obtained in our lab. Moreover, our modelistic approach appears to be applicable also to different systems of high practical importance, like the complex polymerization chain of reactions leading to the formation of lignin.^40^ A main role in the formation of the precursors is played by the hydrogenolysis reaction, which follows oxidation.^41^ The main conclusions follow.

1 Our results account explicitly for the fundamental role played by the base electrolyte. The latter, in the polymerization process, is acting as a hidden kind of catalyzer. Indeed, the final protonated form  is a true-product, but due to the very large amount of the sulfate anion base electrolyte present in the solution (usually 20–100 times the concentration of the electroactive species), the relevant variation of concentration is negligible.
2 Ab initio MD (DRC) trajectories clearly show that the dimer formation can be directly obtained starting from the *first transition state* structure of each reaction path (C and D). Thus, the role played by intermediate species (sigma complex) and subsequent transition states is actually minor, if not negligible (making their search and analysis more an academic subject than a real point needed to explain the observed experimental results). Moreover, the analysis of the DRC trajectory shows that the dissociating proton distance is a significant reaction coordinate parameter, but the dihedral angle between the proton and the aromatic ring is equally important (compare route D sigma complex dissociation).
3 DRC molecular dynamics data show also the existence, and not negligible role, of “transient interactions” of an electrostatic nature (hydrogen bond) mainly between the sulfate oxygens and hydroxyl protons, which yield oscillations in energy due to transient stray intermediates.
4 The active role of the base electrolyte gives reason for the experimental studies devoted to investigating the so-called “base electrolyte” effect. Indeed, different results in the polymerization process can be expected as a function of the chemical nature of the base electrolyte anion.^42−46^

## Supporting Information Available

Further details are reported concerning the calculation of redox standard potentials, cross-check of B3LYP energy values with MP3 and MP4 results, relaxed scan PES of proton dissociation, and DRC results of auxiliary systems not including the sulfate anion (to integrate the DRC results reported in the main manuscript, transition states energies, and coordinates) (PDF)
