# Supplementary material for: The Fundamental and Underrated Role of the Base Electrolyte in the Polymerization Mechanism. The Resorcinol Case Study
Source: J Phys Chem A. 2020 Dec 22;125(1):34–42. doi: 10.1021/acs.jpca.0c07702 (PMC8016188; doi:10.1021/acs.jpca.0c07702)
Supplement: Supplementary file 1 — jp0c07702_si_001.pdf [file jp0c07702_si_001.pdf]

SUPPORTING INFORMATION

The Fundamental and Underrated Role of the Base Electrolyte in the Polymerization Mechanism. The Resorcinol Case Study.

Marco Bonechi[1], Massimo Innocenti[1], Davide Vanossi[2], Claudio Fontanesi[2]*

[1]*Department of Chemistry, University of Firenze, via della Lastruccia 3, 50019 Sesto Fiorentino, Italy.*

[2]*Department of Engineering 'Enzo Ferrari', University of Modena and Reggio Emilia, Via Vivarelli 10, 41125 Modena, Italy.*

Theoretical calculations offer the possibility for a quantitative determination of some physical and electronical quantity, like the HOMO/LUMO energy gap in polymers.[1–3] In particular, values of standard oxidation potential of resorcinol ($E^0_{(1)/(2)}$ of the (1)/(2) redox couple) can be estimated by using reactions shown in Scheme S1. The relevant standard free Gibbs energy variation $\Delta G^0_{(1)/(2)\ \mathrm{vs\ NHE}}$ is obtained with reference to the NHE reaction[4,5] as show in equation (S1). One determined the $\Delta G^0_{(1)/(2)\ \mathrm{vs\ NHE}}$ value, the standard potential is obtained through the Nernst equation (S2).

$$\Delta G^0_{(1)/(2)\ \mathrm{vs\ NHE}} = \Delta G^0_{(1)/(2)} + \Delta G^0_{H^+/H_2} \qquad \text{(S1)}$$

$$\Delta G^0_{(1)/(2)\ \mathrm{vs\ NHE}} = -nFE^0 \qquad \text{(S2)}$$

A value of 4.36 eV has been assigned to the $\Delta G^0_{H^+/H_2}$ of the couple H^+/H_2 and $\Delta G^0_{(1)/(2)}$ has been calculated as the UB3LYP/cc-pVTZ level of theory using the Barone and Cossi's polarizable conductor model (CPCM) method to involve solute-solvent interaction.[6–9]

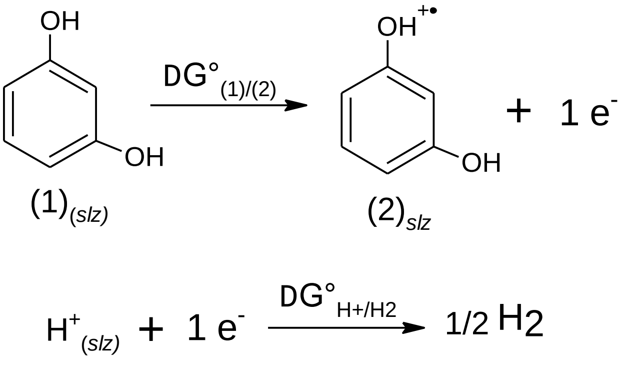

Scheme S1.

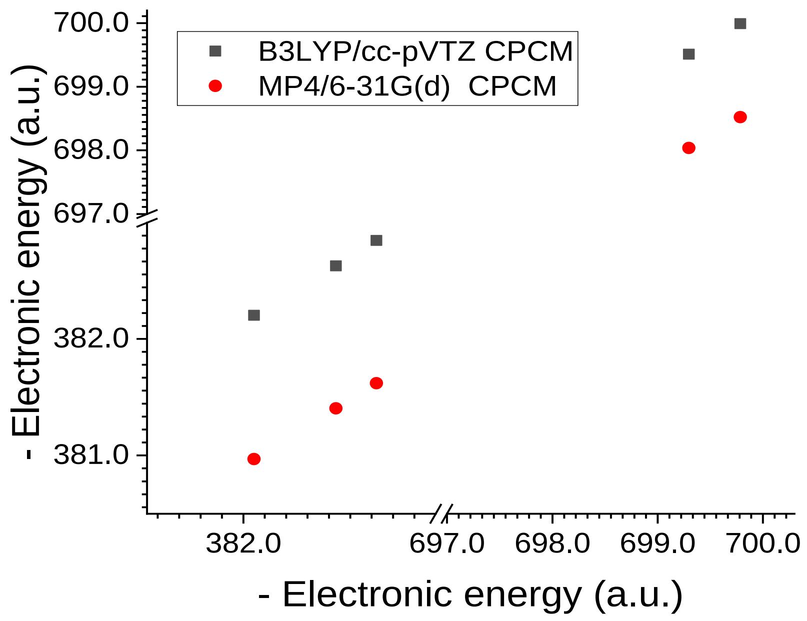

Figure S1a. Abscissa B3LYP/6-31G(d) energy in CPCM solvent. Black squares B3LYP/cc-pVTZ CPCM. Red circles MP4/6-31G(d).

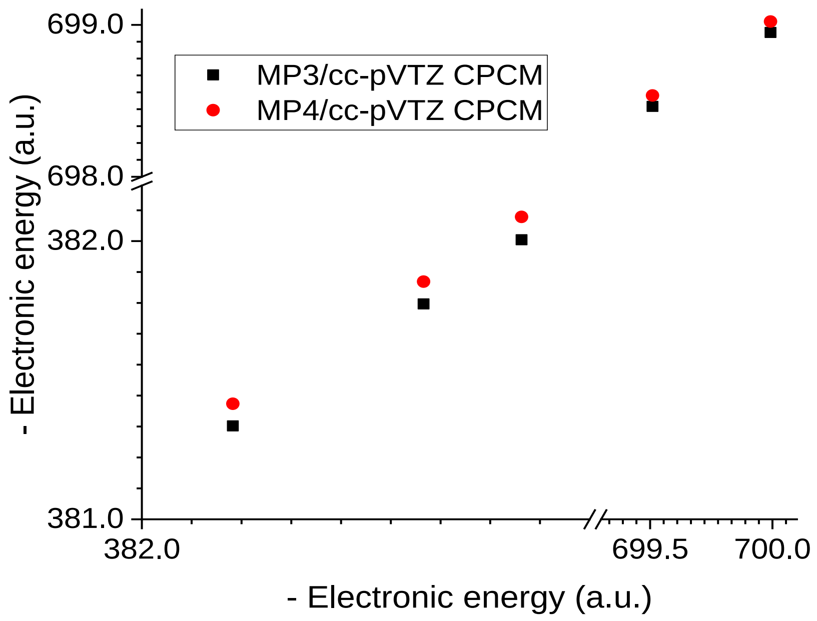

Figure S1b. Abscissa B3LYP/cc-pVZT energy in CPCM solvent. Black squares MP3/cc-pvtz CPCM. Red circles MP4/cc-pVTZ CPCM.

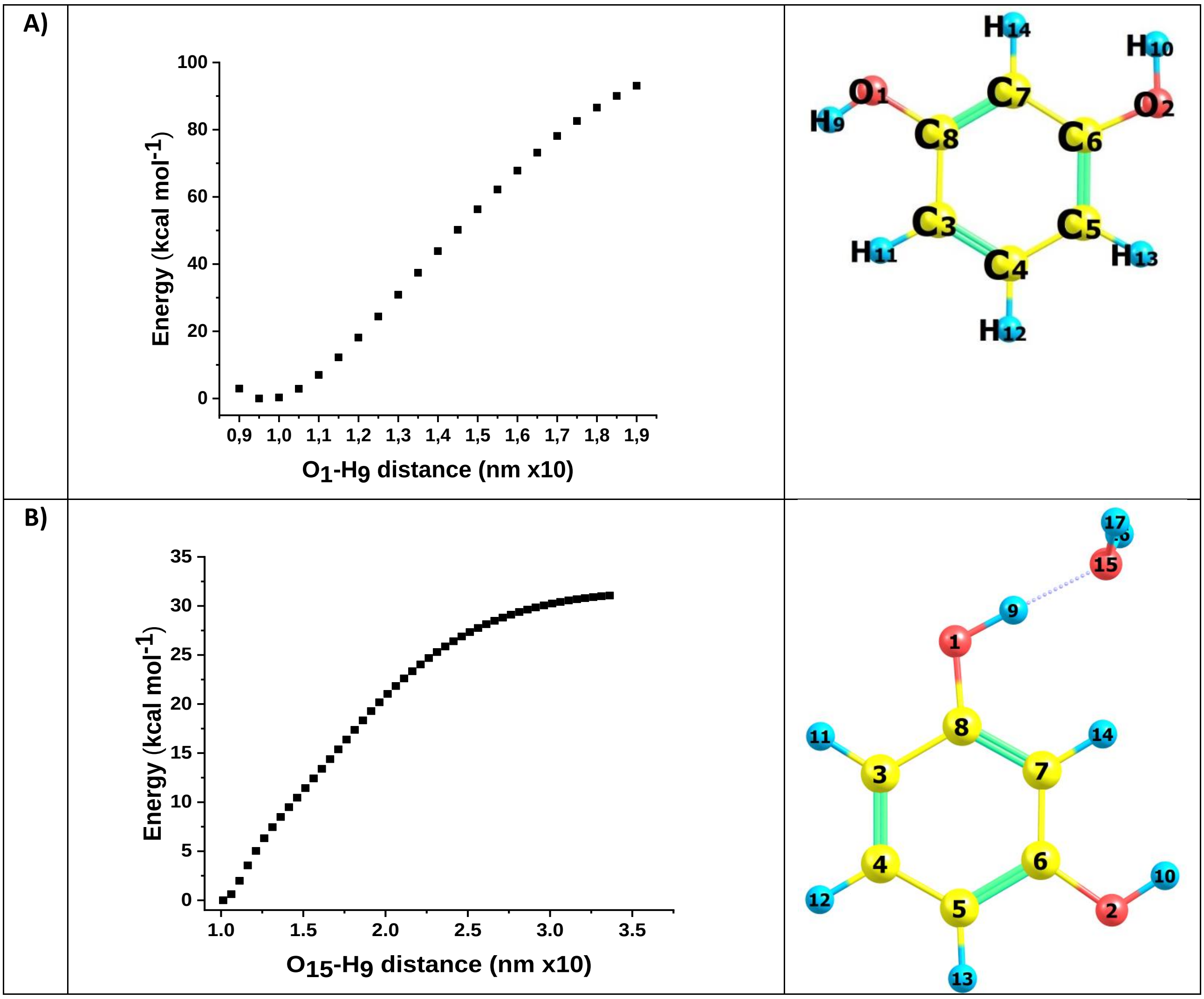

Figure S2. UB3LYP/cc-pVTZ level of theory, using the Barone and Cossi's polarizable conductor model (CPCM).[10] A) relaxed-scan, molecular potential energy vs $O_1 – H_9$ distance for the resorcinol radical cation (2). B) relaxed-scan, molecular potential energy vs $O_1 – H_9$ distance for the resorcinol radical cation (2) plus one water molecule (yielding neutral radical (3) plus H_3O^+).

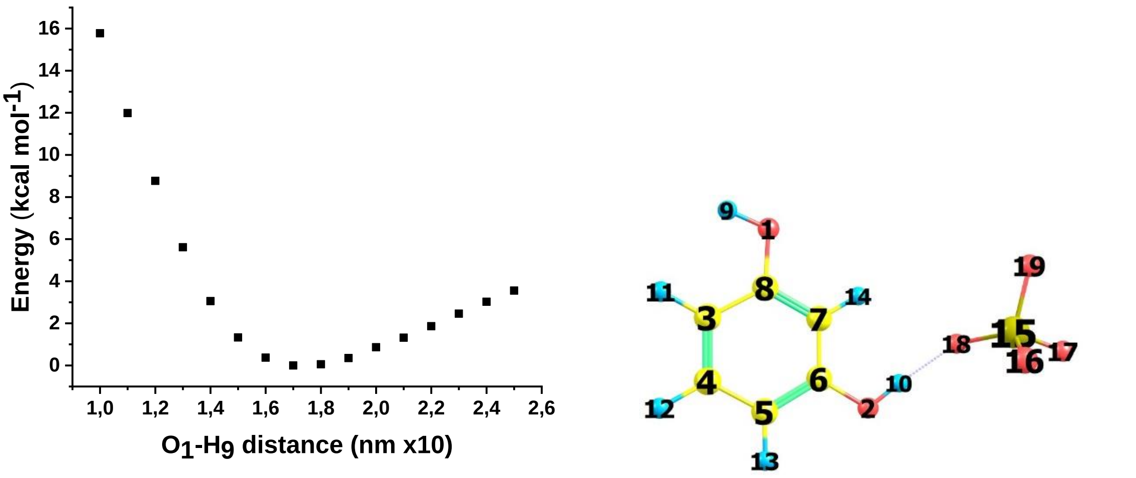

Figure S3. Scan-relax, molecular potential energy vs $O_2 - H_{10}$ distance for the resorcinol radical cation (2) in presence of one molecule of sulphate (electrolytic anion present in solution). UB3LYP/cc-pVTZ level of theory, using the Barone and Cossi's polarizable conductor model (CPCM).[10]

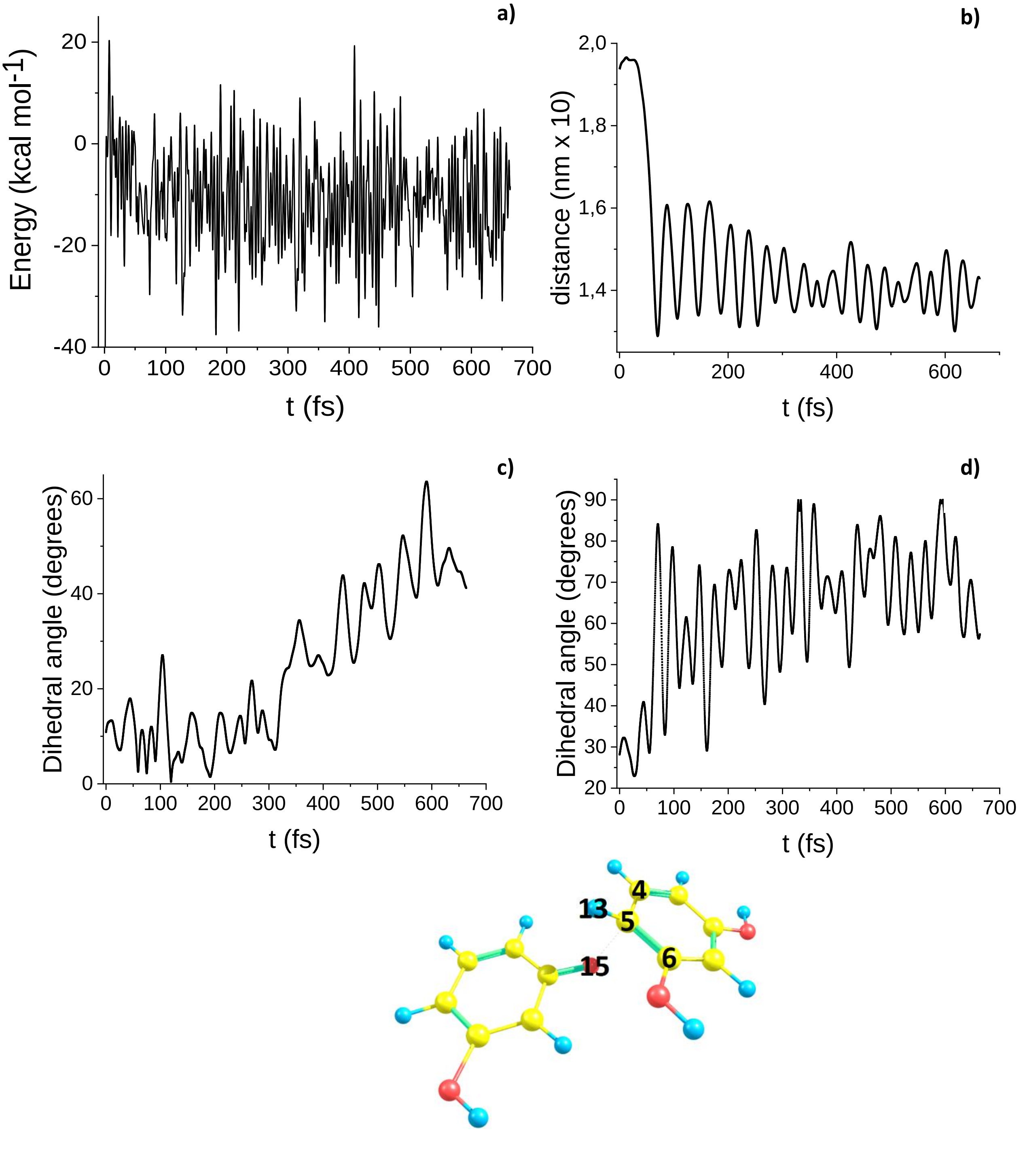

Figure S4. Path (C) DRC results, for TS1(C) that form adduct (4), B3LYP/6-31G(d). a) molecular electronic potential-energy vs time. b) $C_5 - O_{15}$, bond distance, c) dihedral angle between aromatic rings, d) dihedral angle atoms $6 - 4 - 5 - 13$.

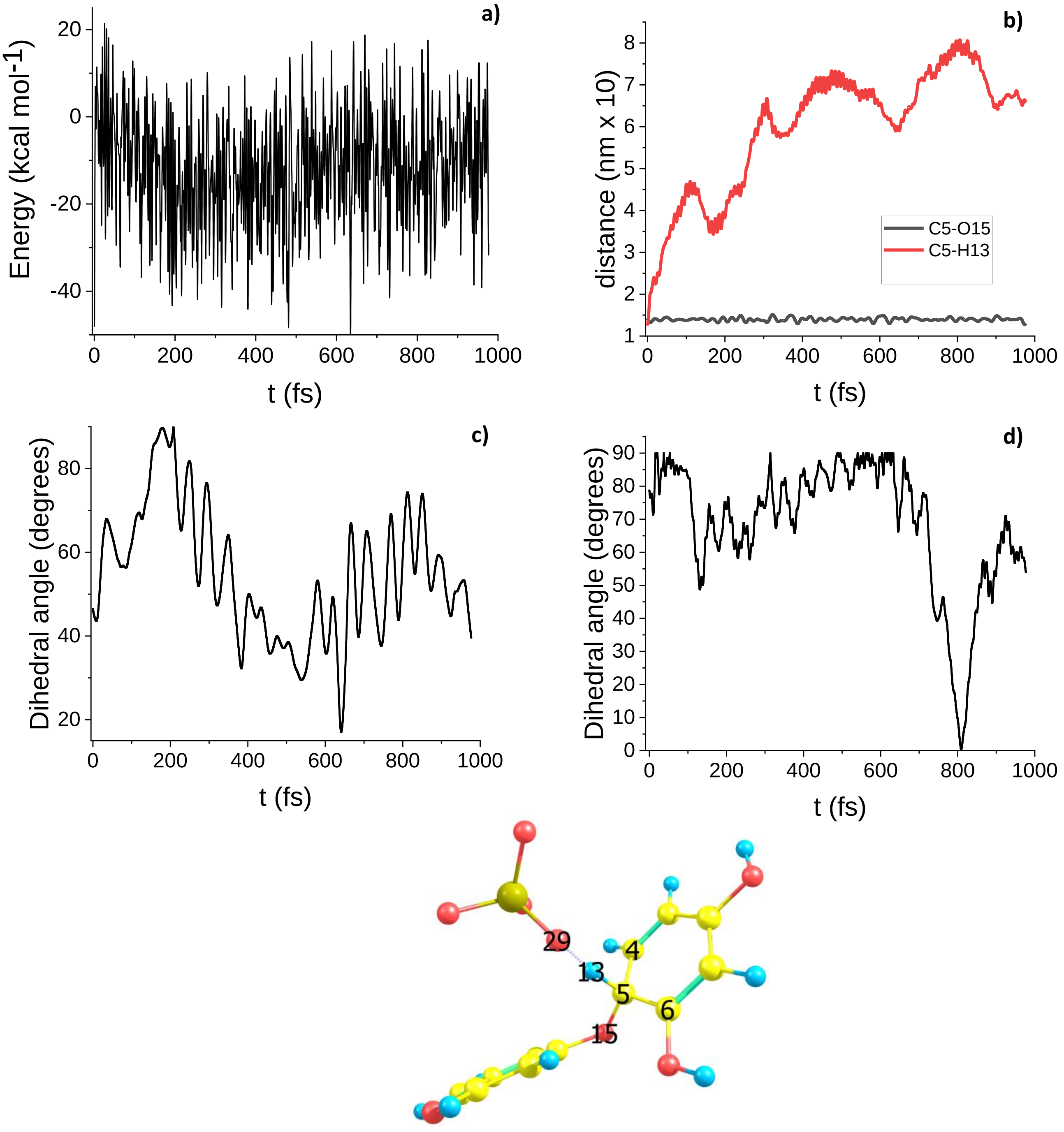

Figure S5. Path (C) DRC results, for TS2(C) that form products (5) and HSO_4^-, B3LYP/6-31G(d). a) molecular electronic potential-energy vs time, b) $C_5 - O_{15}$, $C_5 - H_{13}$ bonds distances, c) dihedral angle between aromatic rings, d) dihedral angle atoms $6 - 4 - 5 - 13$.

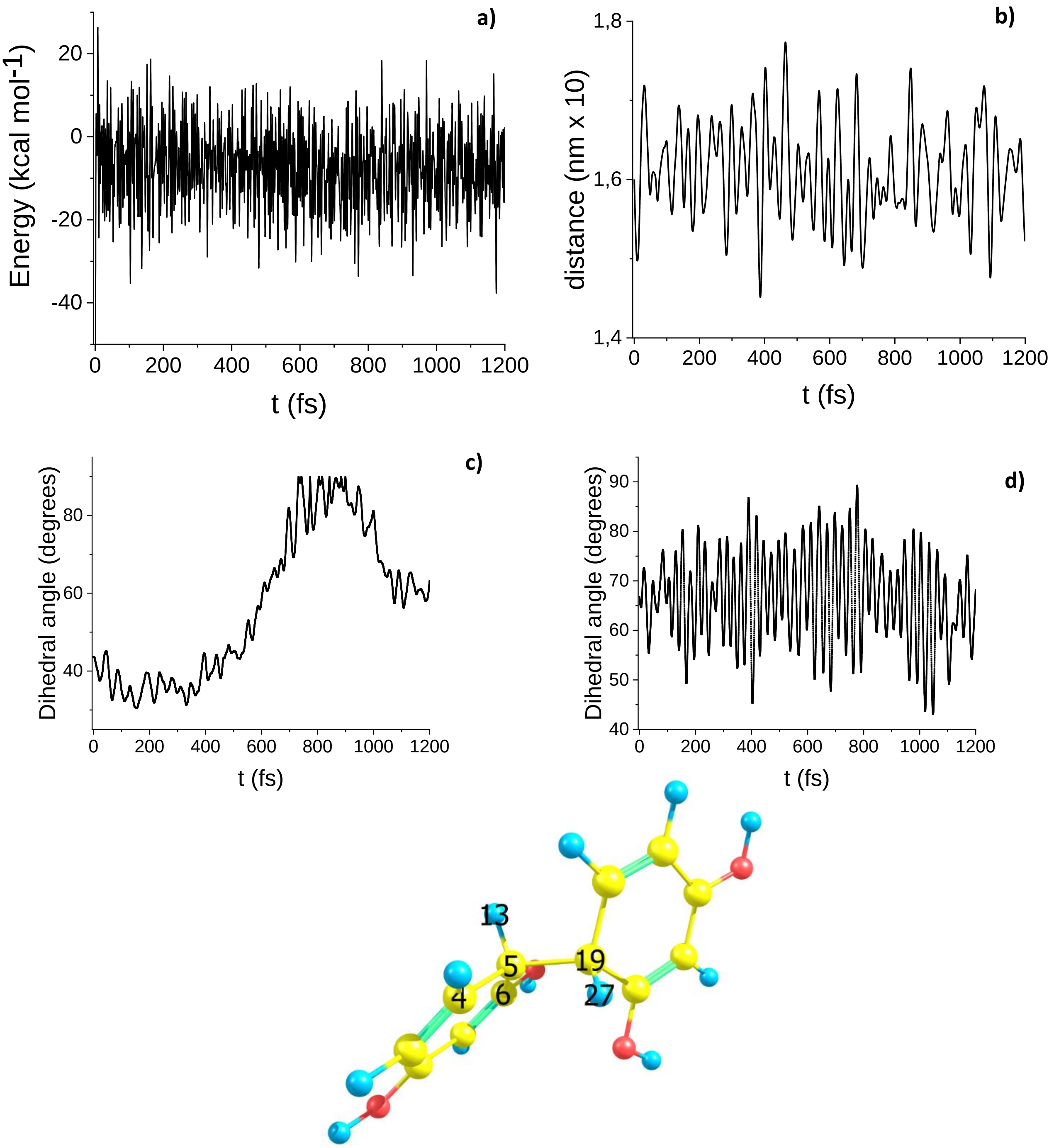

Figure S6. Path (D) DRC results for TS1(D) that form adduct (6), B3LYP/6-31G(d). a) molecular electronic potential-energy vs time. b) $C_5 - C_{19}$ bond distance, c) dihedral angle between aromatic rings, d) dihedral angle between atoms $6 - 4 - 5 - 13$.

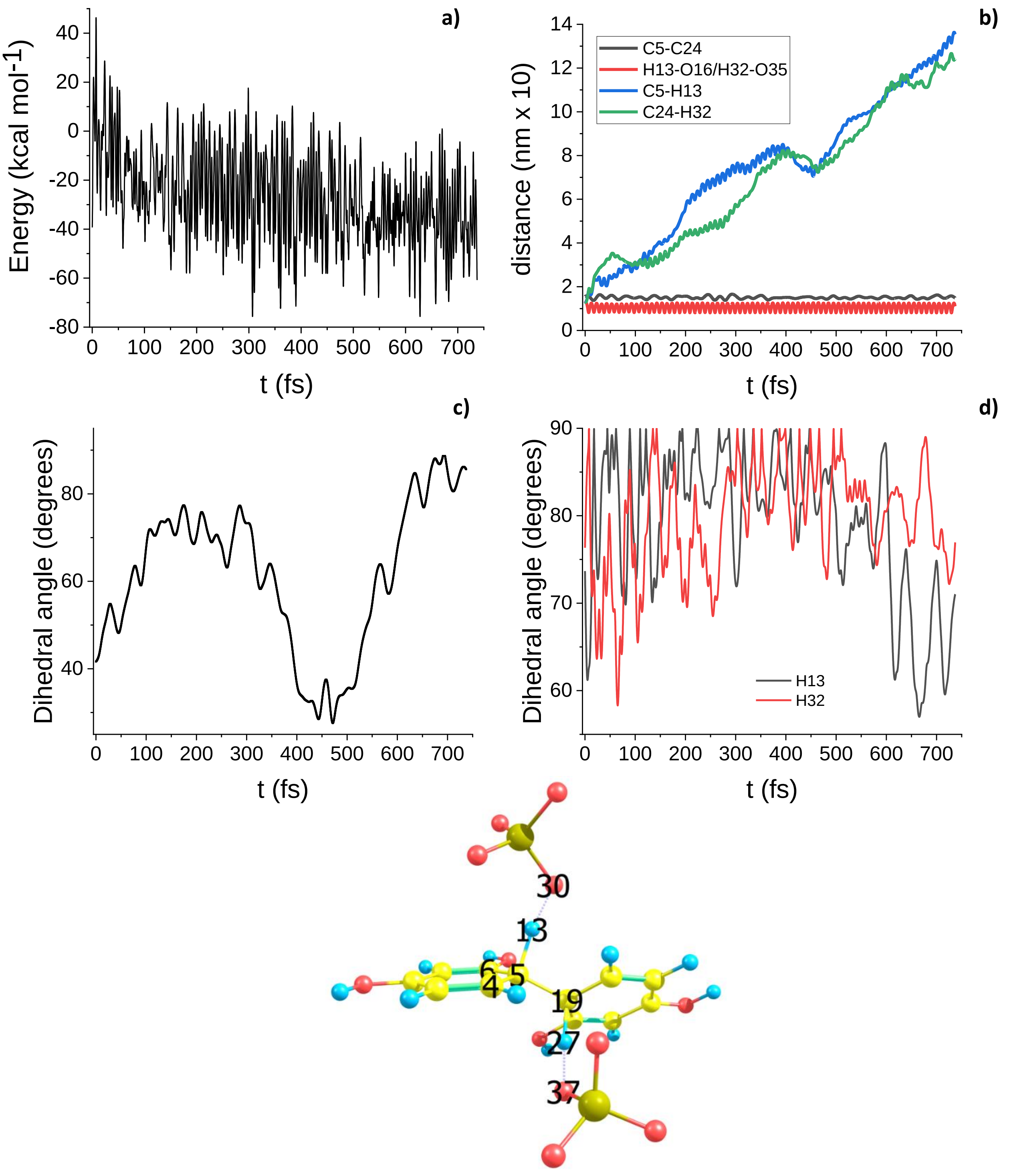

Figure S7. Path (D) DRC results for TS2(D) that form product (7) and two molecules of HSO_4^-, B3LYP/6-31G(d). a) molecular electronic potential-energy vs time. b) $C_5 - C_{19}$, $C_5 - H_{13}$, $C_{19} - H_{27}$, $H_{13} - O_{16}/H_{32} - O_{35}$ bonds distances,, c) dihedral angle between aromatic rings, d) dihedral angle between atoms $6 - 4 - 5 - 13$ (H_{13}) and $18 - 20 - 19 - 27$ (H_{27}).

Calculation specifications

All the theoretical results here reported concerning species in all possible oxidation states and spin multiplicity, are performed in the framework of ab-initio quantum mechanical based methods. Unless otherwise indicated, all calculations were performed using C1 symmetry and unrestricted wavefunction. This by using the GAMESS, Gaussian 16 and Firefly Rev 8.20 (FireFly is partially based on the GAMESS (US)8 source code) programs. Chemcraft is used for visualization purposes, both molecular structures and for ab-initio molecular orbitals display, MacMolPlt served to display DRC trajectories. Original Fortran based codes were created for the extraction of molecular geometrical parameters from DRC calculations, to allow for analysis of angle and bond distance variations as a function of time (available on request to the author C.F.). For all the structures reported as stationary states shown in the PES versus reaction coordinates plot, reaction paths A, B, C and D *vide supra*, molecular geometries are obtained by full-optimization carried out at both the UB3LYP/6-31G* and UB3LYP/cc-pVTZ level of the theory. To account for solute-solvent interaction, geometry optimization is carried out by using the Barone and Cossi's polarizable conductor model (CPCM)[20] method, the latter is based on Tomasi's Polarized Continuum Model (PCM) [21], details of the theoretical solvent parameters: UFF (Universal Force Field) atomic radii, cavity type is scaled VdW (van der Waals Surface) (Alpha=1.100), number of spheres 32, dielectric constants of Water Eps = 78.355300 and Eps(inf) = 1.777849. The stability of all the species is checked by Hessian calculation (vibrational frequency spectrum). In the case of reagents, products and intermediate reaction species all the frequency values are found as real and positive. Transition state search was pursued by analyzing relaxed-scan curves. Transition states feature a single imaginary negative frequency, two negative frequencies are found for the intermediate species which dissociates two protons to yield the final dimer. Ab-initio molecular dynamics, DRC, trajectories (as implemented in the GAMESS and FireFly programs) are started at molecular geometries relevant to stationary points on reaction PES, i.e. transition states and intermediate reaction species. The velocity vector needed to start the molecular dynamics is obtained by projection of Hessian vibrational eigenvectors. In the present DRC results the kinetic energy is partitioned over all normal modes, assigning only the zero-point energy to each normal mode (unless otherwise stated). This trying to minimize the influence of initial geometrical and velocity vector on the molecular dynamic trajectory (an alternative strategies would be to assign arbitrary energy to some selected vibrational modes, for instance the mode leading to dissociation of the proton which corresponds to an imaginary frequency in the transition state).[9] MP3/cc-pVTZ and MP4(SDTQ)/cc-pVTZ single-point energy calculations Figure 1SI are used to cross-check the energy ordering of DFT data (Moller-Plesset post-scf method exploits a completely “different” strategy to

account for electron correlation effects). This is a point of paramount importance in that DRC calculations run on a “wrong” PES would be meaningless.

Peroxo product

Reaction between two (3) radicals would lead to a terminating-polymerization process compound, yielding a peroxo compound (8), Figure S8.

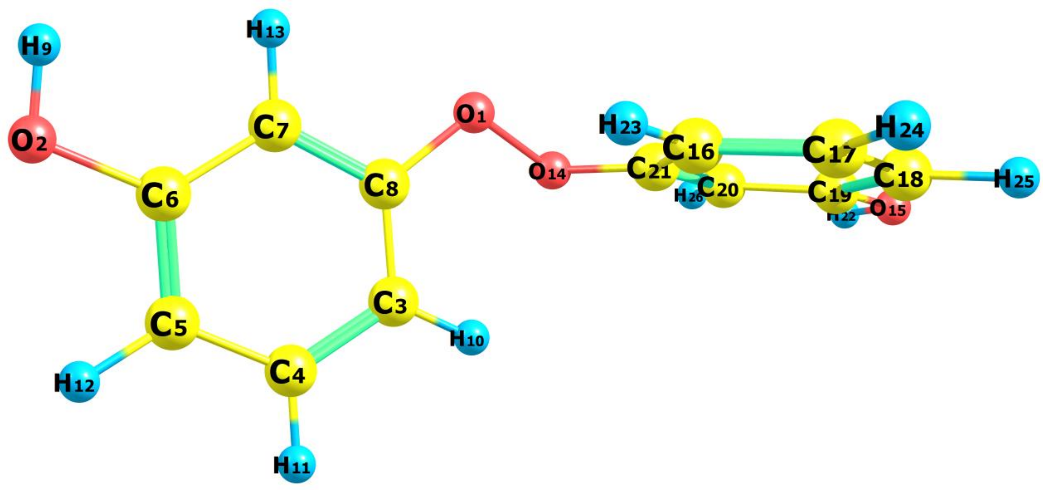

Figure S8. Path (E) product, UB3LYP/CC-pVTZ hessian calculation is characterized by only positive vibrational frequency values. The relevant coordinates here follows.

```
Species (8) 26 centers
symmetry c1
O       -0.545466000      0.469295000     -1.061858000
O       -5.163565000      0.931209000     -0.150417000
C       -1.612425000     -1.156680000      0.433940000
C       -2.799161000     -1.536802000      1.058252000
C       -3.979853000     -0.838010000      0.865993000
C       -3.982877000      0.271633000      0.018831000
C       -2.817081000      0.667799000     -0.627239000
C       -1.645285000     -0.050516000     -0.401970000
H       -5.044226000      1.674989000     -0.752113000
H       -0.700314000     -1.708037000      0.589591000
H       -2.793919000     -2.398552000      1.711876000
H       -4.896688000     -1.136014000      1.353945000
H       -2.808236000      1.518752000     -1.295402000
O        0.545659000     -0.471703000     -1.060828000
O        5.164207000     -0.929898000     -0.149718000
C        1.611836000      1.156274000      0.433327000
C        2.798396000      1.537665000      1.057177000
C        3.979509000      0.839436000      0.865369000
C        3.983138000     -0.270893000      0.019120000
C        2.817514000     -0.668301000     -0.626517000
C        1.645295000      0.049427000     -0.401669000
H        5.045221000     -1.674361000     -0.750640000
H        0.699398000      1.707198000      0.588601000
H        2.792702000      2.399961000      1.710076000
H        4.896202000      1.138425000      1.352982000
H        2.809138000     -1.519769000     -1.294033000
```

Carbon hydrogen, sigma complex, dissociation attempt

Reaction between (2) radical cation, sigma complex, with two SO_4^{2-} anions, Figure S9(a), initial guess geometry, Figure S9(b), optimized geometry, UB3LYP/6-31g(d).

(a)	(b)

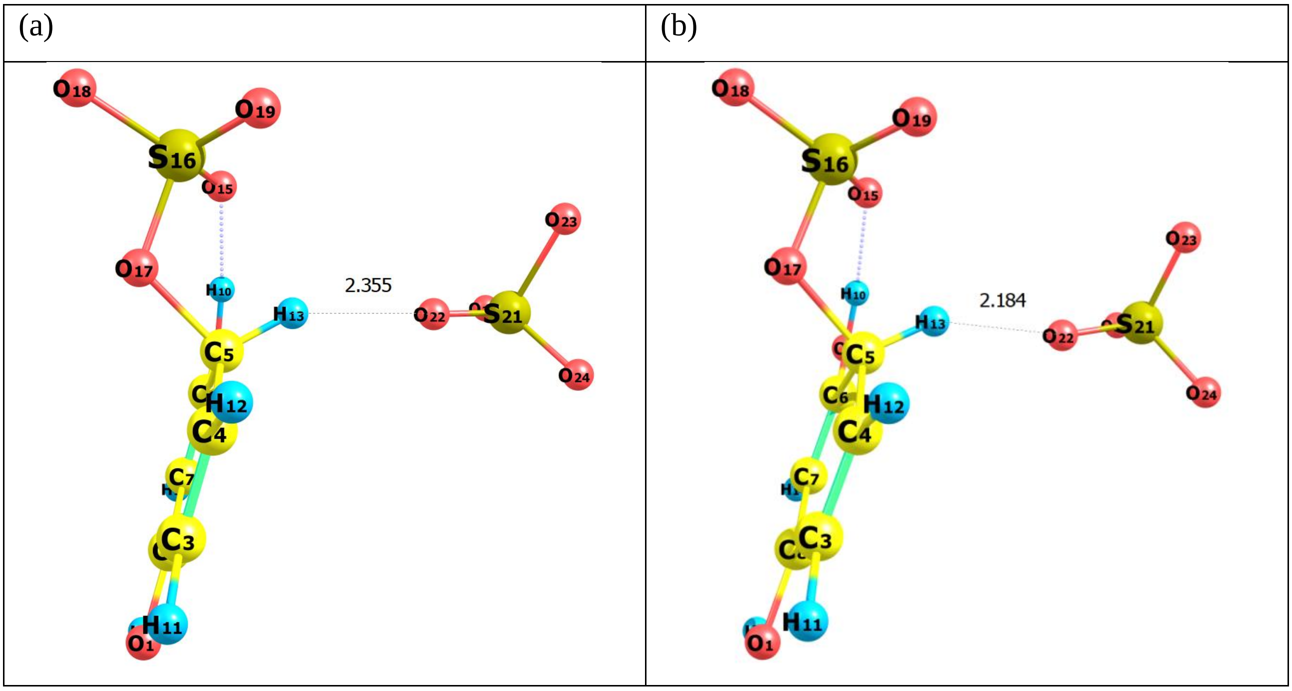

Figure S9. Probing the carbon proton dissociation of (1), with two explicit SO_4^{2-} anions. The relevant coordinates here follows.

```
24 centers
symmetry c1
O        3.492648000     -3.644068000     -0.088410000
O        1.138430000     -0.079746000      2.088670000
C        2.386678000     -2.003274000     -1.405362000
C        1.631544000     -0.866333000     -1.525304000
C        1.185688000     -0.070028000     -0.350405000
C        1.559072000     -0.694362000      0.961213000
C        2.312492000     -1.840418000      1.044659000
C        2.737036000     -2.503635000     -0.129686000
H        3.653221000     -3.886749000      0.838264000
H        0.757228000      0.808195000      1.849013000
H        2.711355000     -2.553743000     -2.284228000
H        1.340514000     -0.495069000     -2.503417000
H        0.105861000      0.124234000     -0.381914000
H        2.572941000     -2.233362000      2.024994000
O        0.281950000      2.321080000      1.222003000
S        0.915059000      2.603539000     -0.111228000
O        1.868183000      1.283587000     -0.418992000
O        1.906842000      3.696902000     -0.077425000
O       -0.076008000      2.686964000     -1.206698000
O       -3.862054000     -0.870770000      1.423077000
S       -3.452302000     -0.742772000     -0.030453000
O       -1.942162000     -0.585693000     -0.114179000
O       -4.120275000      0.471289000     -0.644154000
```

O	-3.865091000	-1.987981000	-0.789705000

Dimeric quinoid structure

Reaction between two (2) radical cations, leading to a quinoid equilibrium structure.

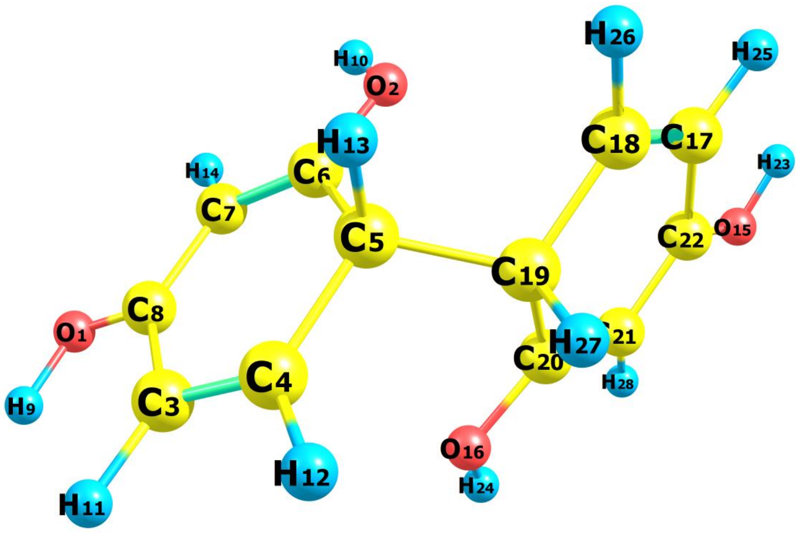

Figure S10. Quinoid equilibrium structure. The relevant coordinates here follows.

```
28 centers
symmetry c1
O       -4.207077000     -0.040022000     -1.187241000
O       -0.144443000      2.117969000     -0.307771000
C       -2.854509000     -0.863018000      0.609196000
C       -1.724412000     -0.751925000      1.310787000
C       -0.717732000      0.333169000      1.091492000
C       -1.079832000      1.223786000     -0.065657000
C       -2.230106000      1.080527000     -0.792721000
C       -3.124307000      0.062757000     -0.456658000
H       -4.772897000     -0.779107000     -0.914786000
H       -0.372341000      2.724071000     -1.029735000
H       -3.578670000     -1.633736000      0.832511000
H       -1.492851000     -1.441602000      2.111607000
H       -0.765546000      1.029067000      1.963316000
H       -2.460557000      1.737634000     -1.618924000
O        4.231129000     -0.125414000     -1.179541000
O        0.071209000     -2.045870000     -0.204136000
C        2.892742000      0.877414000      0.533337000
C        1.757357000      0.852286000      1.235436000
C        0.726941000     -0.223778000      1.098708000
C        1.051964000     -1.190868000     -0.003063000
C        2.218489000     -1.152257000     -0.717537000
C        3.140740000     -0.137525000     -0.454302000
H        4.810321000      0.622836000     -0.968537000
H        0.251261000     -2.675303000     -0.918605000
H        3.645574000      1.633970000      0.708969000
H        1.555904000      1.595421000      1.996173000
H        0.796382000     -0.851739000      2.022186000
H        2.432865000     -1.878233000     -1.488774000
```

Transition states coordinates and total energies.

TS(B) SCF Done: E(UB3LYP) = -1082.13982041 a.u.

19

symmetry c1

O	-2.979863000	2.420133000	-0.086702000
O	-0.354754000	-1.570192000	-0.267550000
C	-4.059158000	0.260056000	0.174101000
C	-3.910414000	-1.131596000	0.219809000
C	-2.669354000	-1.732340000	0.069173000
C	-1.523070000	-0.932150000	-0.134442000
C	-1.662534000	0.468643000	-0.182219000
C	-2.920265000	1.046801000	-0.028627000
H	-3.901636000	2.689287000	0.031596000
H	0.692971000	-0.510367000	-0.590857000
H	-5.040469000	0.722774000	0.294672000
H	-4.792774000	-1.752177000	0.376347000
H	-2.544955000	-2.810247000	0.103040000
H	-0.790869000	1.098337000	-0.336749000
S	3.015096000	0.153223000	0.130234000
O	3.705705000	-0.709162000	-0.962924000
O	3.703818000	1.439619000	0.316823000
O	1.714068000	0.318230000	-0.694647000
O	2.786647000	-0.619335000	1.361429000

TS1(C) SCF Done: E(UB3LYP) = -764.834424807 a.u.

27

symmetry c1

O	-4.811729000	0.154160000	-0.885812000
O	-0.829873000	2.041789000	0.800942000
C	-3.190641000	-1.296634000	0.137807000

C	-1.979438000	-1.411075000	0.738044000
C	-1.127268000	-0.273098000	0.889830000
C	-1.638953000	1.023460000	0.515615000
C	-2.869638000	1.139100000	-0.098061000
C	-3.640179000	-0.011356000	-0.292603000
H	-5.294325000	-0.678713000	-0.975305000
H	-1.198276000	2.885633000	0.506480000
H	-3.827400000	-2.157393000	-0.008392000
H	-1.628242000	-2.374989000	1.076224000
H	-0.362284000	-0.277376000	1.647557000
H	-3.270343000	2.088667000	-0.421183000
O	0.044107000	-0.540549000	-0.629215000
O	4.226697000	1.747908000	-0.221671000
C	1.996702000	-1.761339000	-0.076346000
C	3.369683000	-1.769344000	0.074120000
C	4.087302000	-0.578108000	0.024421000
C	3.429252000	0.650926000	-0.189118000
C	2.058701000	0.676094000	-0.356343000
C	1.315496000	-0.540281000	-0.333649000
H	3.700410000	2.543094000	-0.366344000
H	1.418017000	-2.672582000	-0.027072000
H	3.888300000	-2.703165000	0.238093000
H	5.161212000	-0.561723000	0.145099000
H	1.513199000	1.592006000	-0.531409000

TS2(C) SCF Done: E(UB3LYP) = -1464.40267622 a.u.

32

symmetry c1

O	-4.841658000	-0.676745000	-0.278127000
O	-0.687076000	-2.439330000	-1.619657000
C	-2.985642000	-0.509508000	1.228884000
C	-1.667401000	-0.720410000	1.442891000
C	-0.785306000	-1.161905000	0.372630000
C	-1.443753000	-1.754841000	-0.773018000
C	-2.792164000	-1.512416000	-1.007134000
C	-3.550566000	-0.887587000	-0.023199000
H	-5.282780000	-0.258023000	0.474802000
H	-1.188176000	-2.686849000	-2.410287000
H	-3.626099000	-0.098241000	1.996043000
H	-1.211893000	-0.456166000	2.387357000
H	-0.591310000	-0.030119000	-0.120592000
H	-3.282486000	-1.870131000	-1.900250000
O	0.367193000	-1.864386000	0.808477000
O	3.732133000	0.241414000	-1.956747000
C	2.536909000	-1.451861000	1.640362000
C	3.846518000	-1.022886000	1.443570000
C	4.240667000	-0.457748000	0.240328000
C	3.306132000	-0.322358000	-0.786813000
C	1.998311000	-0.763884000	-0.616696000
C	1.619418000	-1.327460000	0.602286000
H	3.000732000	0.289584000	-2.582988000
H	2.226988000	-1.890876000	2.578515000
H	4.565014000	-1.130110000	2.244992000
H	5.253571000	-0.116343000	0.080450000
H	1.277768000	-0.667575000	-1.415961000
S	-0.318805000	2.491345000	0.027777000

O	-0.544139000	1.161234000	-0.744159000
O	0.908858000	3.134118000	-0.510139000
O	-1.505780000	3.358710000	-0.213144000
O	-0.176930000	2.136300000	1.468363000

TS1(D) SCF Done: E(UB3LYP) = -765.289440253 a.u.

28

symmetry c1

O	-4.656804000	-0.393218000	0.166014000
O	-0.381082000	-1.380992000	-1.422275000
C	-2.958798000	1.263011000	0.453207000
C	-1.680234000	1.613314000	0.262330000
C	-0.685347000	0.721086000	-0.397388000
C	-1.239738000	-0.607389000	-0.795458000
C	-2.554130000	-0.936661000	-0.610600000
C	-3.409723000	-0.026759000	0.016729000
H	-5.194532000	0.286016000	0.601681000
H	-0.771322000	-2.226745000	-1.692187000
H	-3.661350000	1.929453000	0.932905000
H	-1.341328000	2.589663000	0.577317000
H	-0.444717000	1.203625000	-1.356375000
H	-2.948888000	-1.885048000	-0.945167000
O	4.641584000	-0.380769000	-0.242803000
O	0.423522000	-1.387319000	1.476862000
C	2.935970000	1.279946000	-0.457437000
C	1.662414000	1.624164000	-0.227363000
C	0.681567000	0.716756000	0.434798000
C	1.254390000	-0.610111000	0.817226000
C	2.561937000	-0.935074000	0.585428000

C	3.399290000	-0.015806000	-0.053150000
H	5.169659000	0.305716000	-0.679444000
H	0.835821000	-2.223081000	1.746321000
H	3.621117000	1.951643000	-0.953987000
H	1.308218000	2.599069000	-0.528845000
H	0.434753000	1.173826000	1.404267000
H	2.969233000	-1.884620000	0.901190000

TS2(D) SCF Done: E(UB3LYP) = -2164.36775761 a.u.

38

symmetry c1

O	2.698090000	-3.687983000	1.679430000
O	1.457519000	0.778268000	2.467505000
C	1.242095000	-2.606725000	0.098908000
C	0.555328000	-1.488186000	-0.238407000
C	0.579861000	-0.275994000	0.543070000
C	1.360262000	-0.349684000	1.757785000
C	2.071116000	-1.483940000	2.101445000
C	2.002586000	-2.612677000	1.289883000
H	2.601456000	-4.410222000	1.043053000
H	2.040100000	0.657418000	3.229339000
H	1.208805000	-3.489730000	-0.525726000
H	-0.079574000	-1.497625000	-1.116019000
H	1.354176000	0.557763000	-0.102407000
H	2.694993000	-1.500007000	2.984903000
S	3.322595000	1.056866000	-1.450279000

O	1.962714000	1.505954000	-0.813920000
O	3.393307000	1.756741000	-2.760889000
O	4.399419000	1.498019000	-0.520036000
O	3.259821000	-0.426928000	-1.589994000
O	-2.960406000	4.053480000	0.758903000
O	-1.176240000	0.240728000	2.841726000
C	-1.699535000	2.501617000	-0.589549000
C	-0.957031000	1.367246000	-0.623528000
C	-0.759041000	0.519606000	0.527504000
C	-1.302191000	1.029464000	1.768337000
C	-2.035314000	2.194892000	1.816045000
C	-2.236419000	2.933050000	0.649288000
H	-3.059722000	4.485618000	-0.099894000
H	-1.623399000	0.621870000	3.608610000
H	-1.863077000	3.089219000	-1.483613000
H	-0.530261000	1.038870000	-1.560655000
H	-1.645908000	-0.416747000	0.349330000
H	-2.484373000	2.528508000	2.742170000
S	-3.124345000	-1.563887000	-1.235053000
O	-2.635682000	-1.303883000	0.224910000
O	-3.343704000	-3.038669000	-1.326258000
O	-4.374621000	-0.779984000	-1.426178000
O	-2.019658000	-1.086318000	-2.123756000

References

(1) Morvillo, P.; Parenti, F.; Diana, R.; Fontanesi, C.; Mucci, A.; Tassinari, F.; Schenetti, L. A Novel Copolymer from Benzodithiophene and Alkylsulfanyl-Bithiophene: Synthesis, Characterization and Application in Polymer Solar Cells. *Solar Energy Materials and Solar Cells* **2012**, *104*, 45–52. https://doi.org/10.1016/j.solmat.2012.04.044.

(2) Morvillo, P.; Diana, R.; Fontanesi, C.; Ricciardi, R.; Lanzi, M.; Mucci, A.; Tassinari, F.; Schenetti, L.; Minarini, C.; Parenti, F. Low Band Gap Polymers for Application in Solar Cells: Synthesis and Characterization of Thienothiophene-Thiophene Copolymers. *Polym. Chem.* **2013**. https://doi.org/10.1039/C3PY01618H.

(3) Parenti, F.; Ricciardi, R.; Diana, R.; Morvillo, P.; Fontanesi, C.; Tassinari, F.; Schenetti, L.; Minarini, C.; Mucci, A. Polymers for Application in Organic Solar Cells: Bithiophene Can Work Better than Thienothiophene When Coupled to Benzodithiophene. *J. Polym. Sci. Part A: Polym. Chem.* **2015**, n/a-n/a. https://doi.org/10.1002/pola.28014.

(4) Trasatti, S. Structuring of the Solvent at Metal/Solution Interfaces and Components of the Electrode Potential. *Journal of Electroanalytical Chemistry and Interfacial Electrochemistry* **1983**, *150* (1–2), 1–15. https://doi.org/10.1016/S0022-0728(83)80183-1.

(5) Trasatti, S. The “Absolute” Electrode Potential—the End of the Story. *Electrochimica Acta* **1990**, *35* (1), 269–271. https://doi.org/10.1016/0013-4686(90)85069-Y.

(6) Winget, P.; Weber, E. J.; Cramer, C. J.; Truhlar, D. G. Computational Electrochemistry: Aqueous One-Electron Oxidation Potentials for Substituted Anilines. *Phys. Chem. Chem. Phys.* **2000**, *2* (6), 1231–1239.

(7) Winget, P.; Cramer, C. J.; Truhlar, D. G. Computation of Equilibrium Oxidation and Reduction Potentials for Reversible and Dissociative Electron-Transfer Reactions in Solution. *Theoretical Chemistry Accounts* **2004**, *112* (4). https://doi.org/10.1007/s00214-004-0577-0.

(8) Fontanesi, C. Theoretical Study of the Dissociative Process of the 4-Chlorotoluene Radical Anion. *Journal of Molecular Structure: THEOCHEM* **1997**, *392*, 87–94. https://doi.org/10.1016/S0166-1280(97)90379-1.

(9) Fontanesi, C.; Baraldi, P.; Marcaccio, M. On the Dissociation Dynamics of the Benzyl Chloride Radical Anion. An Ab Initio Dynamic Reaction Coordinate Analysis Study. *Journal of Molecular Structure: THEOCHEM* **2001**, *548* (1–3), 13–20. https://doi.org/10.1016/S0166-1280(00)00863-0.

(10) Barone, V.; Cossi, M. Quantum Calculation of Molecular Energies and Energy Gradients in Solution by a Conductor Solvent Model. *J. Phys. Chem. A* **1998**, *102* (11), 1995–2001. https://doi.org/10.1021/jp9716997.
